# A seed germination transcriptomic study contrasting two soybean genotypes that differ in terms of their tolerance to the deleterious impacts of elevated temperatures during seed fill

**DOI:** 10.1186/s13104-019-4559-7

**Published:** 2019-08-19

**Authors:** Jason D. Gillman, Jessica J. Biever, Songqing Ye, William G. Spollen, Scott A. Givan, Zhen Lyu, Trupti Joshi, James R. Smith, Felix B. Fritschi

**Affiliations:** 1 USDA-ARS, Plant Genetics Research Unit, 205 Curtis Hall, University of Missouri, Columbia, MO 65211 USA; 2 Divisions of Plant Science, University of Missouri-Columbia, Columbia, MO 65211 USA; 3 Informatics Research Core Facility, University of Missouri-Columbia, Columbia, MO 65211 USA; 4 Bioinformatics and Biostatistics Core, Van Andel Research Institute, University of Missouri-Columbia, Columbia, MO USA; 5 Department of Electrical Engineering and Computer Science, University of Missouri-Columbia, Columbia, MO USA; 6 Health Management and Informatics, MU Informatics Institute, Interdisciplinary Plant Group and Christopher S. Bond Life Science Center, University of Missouri-Columbia, Columbia, MO 65211 USA; 7 USDA-ARS, Crop Genetics Research Unit, Stoneville, MS 38776 USA; 8 Present Address: Metropolitan Community College-Penn Valley, Kansas City, MO USA

**Keywords:** Soybean, Transcriptomic analysis, Seed germination, Temperature stress, Seed development

## Abstract

**Objective:**

Soybean seed development is negatively impacted by elevated temperatures during seed fill, which can decrease seed quality and economic value. Prior germplasm screens identified an exotic landrace able to maintain ~ 95% seed germination under stress conditions that reduce germination dramatically (> 50%) for typical soybean seeds. Seed transcriptomic analysis was performed for two soybean lines (a heat-tolerant landrace and a typical high-yielding adapted line) for dry, mature seed, 6-h imbibed seed and germinated seed. Seeds were produced in two environments: a typical Midwestern field and a heat stressed field located in the Midsouth soybean production region.

**Results:**

Transcriptomic analysis revealed 23–30K expressed genes in each seed tissue sample, and differentially expressed genes (DEGs) with ≥ twofold gene expression differences (at q-value < 0.05) comprised ~ 5–44% of expressed genes. Gene ontology (GO) enrichment analysis on DEGs revealed enrichment in heat-tolerant seeds for genes annotated for general and temperature-specific stress, as well as protein-refolding. DEGs were also clustered in modules using weighted co-expressed gene network analysis, which were examined for enrichment of GO biological process terms. Collectively, our results provide new and valuable insights into this unique form of genetic abiotic stress tolerance and to soybean seed physiological responses to elevated temperatures.

**Electronic supplementary material:**

The online version of this article (10.1186/s13104-019-4559-7) contains supplementary material, which is available to authorized users.

## Introduction

Soybean (*Glycine max* L. Merr.) is a major commodity crop, comprising ~ 34% (~ 36.5 million ha) of crop land in the United States in 2017 (http://www.soystats.com, accessed 3-21-19). The value of the crop is principally derived from the high yield and quality of oil and protein in the seeds. The Midsouth soybean growing region of the United States experiences consistent late-season drought, which has resulted in historically reduced on-farm seed yield and economic return [1, 2]. Although irrigation can at least partially remedy these issues, fuel for pumping water is expensive and long term use of aquifers for agricultural irrigation may be unsustainable [3].

Traditionally, soybean maturity group (MG) 5–7 cultivars were planted in May and June with harvest in October and November. An alternative method, the Early Soybean Production System (ESPS) modifies soybean planting and harvest dates to avoid much of the late season endemic drought [2] by use of cultivars that flower and mature earlier (typically MG 3–4 and early 5 as compared with MG5-7) and by adjusting planting dates to early-to-mid-April with harvest typically occurring in September. The practices of the ESPS in the Midsouth region has increased seed yield and on-farm return on investment [1, 3] under both irrigated and non-irrigated conditions [1].

Soybean has traditionally been considered to be heat-tolerant, with a vegetative optimum temperature of ~ 30 °C [4]. However, the processes of pollination and seed growth/maturation are sensitive to elevated temperatures; the reproductive optimum is a relatively low 22–24 °C [5]. Despite economic and seed yield gains under the ESPS, seed produced in this system are exposed to much higher temperatures [6] during seed fill (≥ 32 °C maximum daytime temperature) than seeds of MG 5–7 cultivars produced in the traditional system. In typical MG 4 cultivars, exposure to such high temperatures during seed development reduces seed quality/germination, increases pathogen infection, and often results in economic loss through seed dockage [3, 7].

Soybean is a self-pollinating species, and modern high-yielding cultivars derive from an extremely limited genetic base; traditional breeding has exacerbated this problem [8, 9]. Exotic landraces may contain novel disease and stress resistance genes; a successful screen identified lines that can tolerate the high temperature associated with the ESPS [6]. An unimproved landrace (PI 587982A) has consistent and robust resistance (> 90% germination, near absence of *Phomopsis longicolla* infection). The first United States heat tolerant germplasm release, with tolerance derived from PI 587982A, was recently made by our group [10].

Transcriptomics, enabled by advances in DNA sequencing and computation, is a powerful tool to identify gene expression differences and correlations with genetic/developmental cues or environmental conditions. Detailed studies have generated “transcriptomic atlases” for soybean gene expression [11–15]. However, studies have ignored soybean seed germination, in favor of seed development or vegetative tissues (typically leaves or roots). In this study, we examined three soybean seed germination stages: (1) dry, mature seed; (2) imbibed seed; and (3) germinated seed and contrasted two soybean genotypes which differ in their tolerance to the impact of elevated temperature on seed quality, using seed produced in two environments differing in abiotic stress: (A) a lower temperature, Midwest location; and (B) the high temperature conditions of the ESPS.

## Main text

### Methods

#### Field seed production, seed imbibition and germination measurement, and RNA isolation and RNA sequencing, mapping and statistical analysis

Full details are provided in Additional file 1.

#### GO term enrichment and venn diagrams

GO term enrichment was performed using the tool present on Soybase (https://soybase.org/goslimgraphic_v2/dashboard.php) using DEGs identified through Cuffdiff analysis. Venn diagrams were generated using the Venny tool at http://bioinfogp.cnb.csic.es/tools/venny/index.html and the Venn diagram tool at http://bioinformatics.psb.ugent.be/webtools/Venn/.

#### Whole genome comparative network analysis and gene ontology enrichment of co-expressed gene modules

Modules of genes with highly correlated expression patterns were described using weighted gene co-expression network analysis (WGCNA). We expect these modules to correspond to networks of genes that are co-expressed and thus interact and share biological processes. We constructed unsigned weighted gene co-expression modules using the WGCNA [16] package in R. The blockwiseModules function was run with the Pearson correlation coefficient and a soft thresholding power of 18. The resulting genes modules were named by assigning them different colors arbitrarily. Additionally, we further analyzed each module by conducting significant associations for Gene Ontology (GO) function annotations enrichment analysis (Additional file 5) and used hierarchical clustering to group differentially expressed genes across samples (Additional file 2).

g:GOSt (https://biit.cs.ut.ee/gprofiler/gost) was used to examine modules detected by WGCNA, in order to detect statistically significant enriched GO terms within specific modules, using the Benjamin–Hochberg FDR method at α = 0.05 as significant.

#### qRT-PCR analyses

qRT-PCR analysis was performed as described [17], using the ΔΔCt method [18]. FPKM output was normalized to the cons14 [19] gene (Glyma16g32510) and expressed as log2 ratio for comparison to CuffDuff output.

### Results/discussion

#### Germination assays

**Fig. 1:**
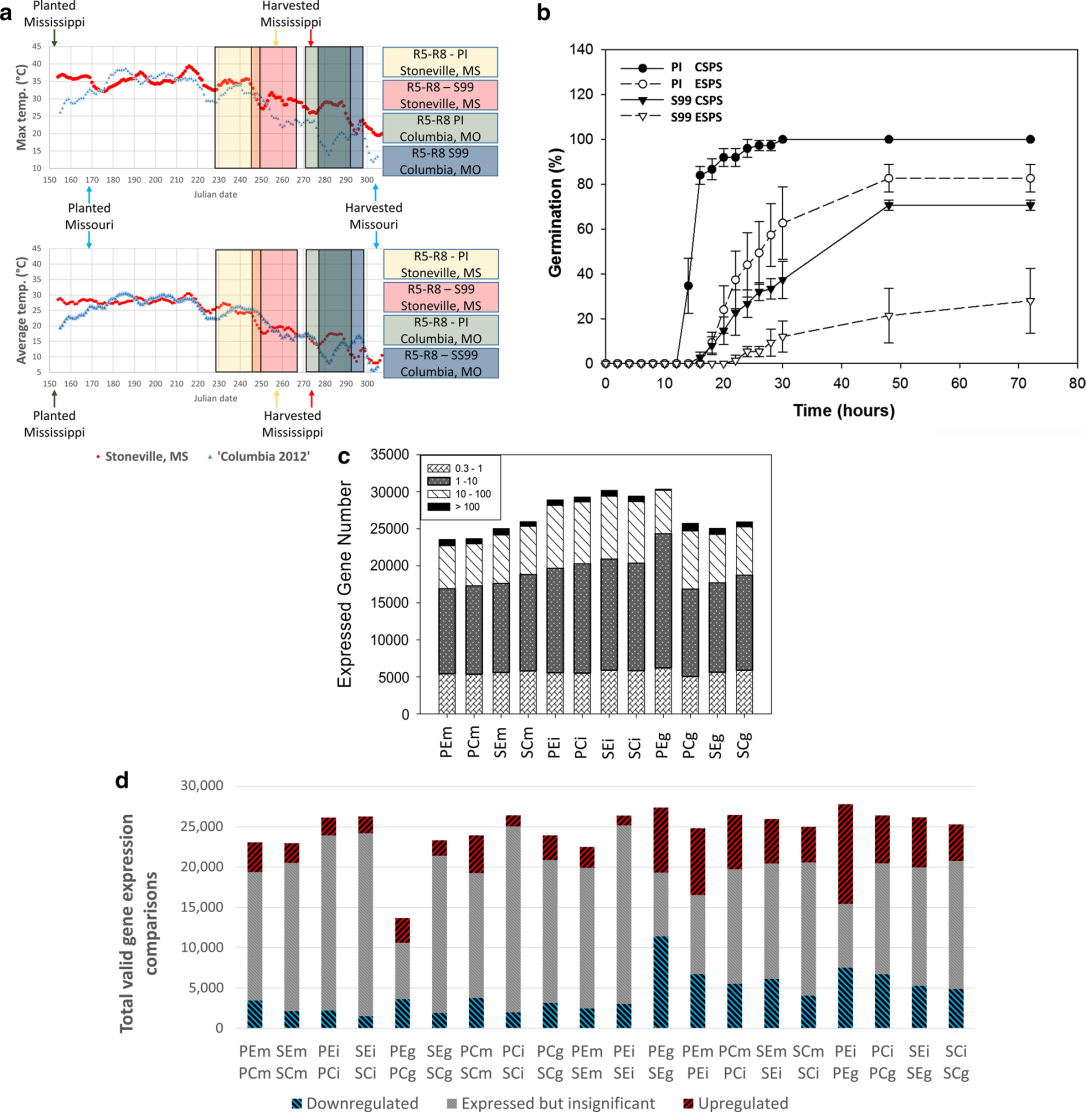
Details on production of soybean seed and RNAseq analysis of seed germination. **a** Weather parameters, planting/harvest dates and approximate period of soybean seed fill (stage R5-R8) for genotypes PI 598982A and S99-11986 produced in Columbia, MO (Conventional Soybean Production System) and Stoneville, MS (Early Soybean Production System). **b** Kinetics of seed germination in this study. Seed of two genotypes, PI 587982A-heat tolerant and S99-11986-heat sensitive, produced in either Columbia, MO (Conventional Soybean Production System) and Stoneville, MS (Early Soybean Production System) were imbibed and germination observed for 72 h (n = 3, 25 seeds per replicated). The mean of three replicates is plotted and error bars indicate standard deviation. **c** Total genes expressed in each sample with an average FPKM ≥ 0.3. **d** Distribution of differential gene expression amongst 20 different comparisons. Upregulation indicates higher level of expression in the top sample as compared to the bottom sample. The names indicated in the horizontal axis for **c** and **d** are three-letter codes which indicates genotype (P = PI 587982A, S = S99-11986), location seed were produced (C = CSPS, E = ESPS), and tissue (M = dry, mature seed; I = 6-h imbibed seed; G = germinated seed)

We examined germination kinetics for two soybean genotypes: (1) a heat-tolerant soybean plant introduction line (PI 587982A) henceforth referred to as “PI”; and (2) S99-11986, a conventional high yielding improved line [20], comparable to cultivars commonly grown in the Midsouth and Midwest regions, henceforth referred to as “SG”. Seed to be germinated were produced (Fig. 1a) in one of two environments: (1) a location with endemic high temperature stress associated with the Early Soybean Production System (henceforth refered to as ESPS—Stoneville, MS; Fig. 1a); or (2) a less stressful Conventional Soybean Production System (CSPS—Columbia, MO; Fig. 1a).

Seed of the PI line were found to germinate much more rapidly than those of the SG line in both environments (Fig. 1b), and PI seed from both unstressed and heat-stressed locations germinated with very high efficiency (> 80%, Fig. 1a). In contrast, only 75% of CSPS-produced seed from SG germinated by the end of 72 h. A dramatic reduction in germination was noted for SG seed produced under the heat-stress of the ESPS (~ 30% germination at 72 h, Fig. 1b). Our germination results are concordant with our previous metabolic study [21].

We then selected three stages (Fig. 1b, Table 1) to obtain transcriptomic data: (1) mature, dry seed; (2) 6-h imbibed seed; and (3) germinated seed with emerged radicle for each genotype grown in both environments (Fig. 1b, Table 1). It is important to note that the time from imbibition to germination varied between genotype/environments (Fig. 1b). Three biological replicates (each consisting of 5 seed) per genotype/condition/timepoint were used for analysis to quantify gene expression. The number of genes expressed (FPKM > 0.3) in each sample ranged from 23,560 to 30,349 (Fig. 1c, Table 1).

**Table 1:** RNAseq sample details

NCBI SRA accession#	PI#	Stress condition	Seed tissue	Bio rep	Illumina index	Barcode	Mapped reads (Mil)	For QS	Rev QS
SAMN10588589	PI587982A	CSPS	Mature	1	4	TGACCA	833.2	38.2	37.6
SAMN10588590	PI587982A	CSPS	Mature	2	16	CCGTCC	446.9	38.2	37.4
SAMN10588591	PI587982A	CSPS	Mature	3	14	AGTTCC	444.3	38.2	37.3
SAMN10588592	S99-11986	CSPS	Mature	1	19	GTGAAA	294.4	38.1	36.9
SAMN10588593	S99-11986	CSPS	Mature	2	13	AGTCAA	346.8	38	36
SAMN10588594	S99-11986	CSPS	Mature	3	15	ATGTCA	334.2	38.1	36.9
SAMN10588595	PI587982A	CSPS	Imbibed	1	14	AGTTCC	362.4	38.1	36.8
SAMN10588596	PI587982A	CSPS	Imbibed	2	4	TGACCA	329.5	38.1	37
SAMN10588597	PI587982A	CSPS	Imbibed	3	16	CCGTCC	311.4	38.5	37.4
SAMN10588598	S99-11986	CSPS	Imbibed	1	5	ACAGTG	307.4	38.6	37.3
SAMN10588599	S99-11986	CSPS	Imbibed	2	7	CAGATC	269.1	38.5	37.1
SAMN10588600	S99-11986	CSPS	Imbibed	3	18	GTCCGC	305.3	38.5	37.4
SAMN10588601	PI587982A	CSPS	Germinated	1	7	CAGATC	910.2	38	36.7
SAMN10588602	PI587982A	CSPS	Germinated	2	18	GTCCGC	316.7	38	36.8
SAMN10588603	PI587982A	CSPS	Germinated	3	5	ACAGTG	448.8	38.1	36.8
SAMN10588604	S99-11986	CSPS	Germinated	1	18	GTCCGC	377.2	38.1	37.3
SAMN10588605	S99-11986	CSPS	Germinated	2	5	ACAGTG	387.2	38.2	37.4
SAMN10588606	S99-11986	CSPS	Germinated	3	7	CAGATC	401.5	38.1	37
SAMN10588607	PI587982A	ESPS	Mature	1	6	GCCAAT	375.4	38.2	37.3
SAMN10588608	PI587982A	ESPS	Mature	2	2	CGATGT	481.8	38.2	37.4
SAMN10588609	PI587982A	ESPS	Mature	3	12	CTTGTA	469.1	38	36.4
SAMN10588610	S99-11986	ESPS	Mature	1	15	ATGTCA	388.3	38.2	37.2
SAMN10588611	S99-11986	ESPS	Mature	2	19	GTGAAA	396.9	38.2	37.3
SAMN10588612	S99-11986	ESPS	Mature	3	13	AGTCAA	429.2	38.2	37.4
SAMN10588613	PI587982A	ESPS	Imbibed	1	12	CTTGTA	229.7	38.5	37.4
SAMN10588614	PI587982A	ESPS	Imbibed	2	6	GCCAAT	332.0	38.6	37.5
SAMN10588615	PI587982A	ESPS	Imbibed	3	2	CGATGT	306.9	38.6	37.5
SAMN10588616	S99-11986	ESPS	Imbibed	1	16	CCGTCC	380.1	38	36.4
SAMN10588617	S99-11986	ESPS	Imbibed	2	14	AGTTCC	287.2	38.6	37.5
SAMN10588618	S99-11986	ESPS	Imbibed	3	4	TGACCA	297.2	38.6	37.3
SAMN10588619	PI587982A	ESPS	Germinated	1	13	AGTCAA	229.0	38.6	37.5
SAMN10588620	PI587982A	ESPS	Germinated	2	15	ATGTCA	314.3	38.6	37.6
SAMN10588621	PI587982A	ESPS	Germinated	3	19	GTGAAA	260.3	38.6	37.6
SAMN10588622	S99-11986	ESPS	Germinated	1	2	CGATGT	499.9	38.1	37
SAMN10588623	S99-11986	ESPS	Germinated	2	12	CTTGTA	290.2	38.2	36.9

**Fig. 2:**
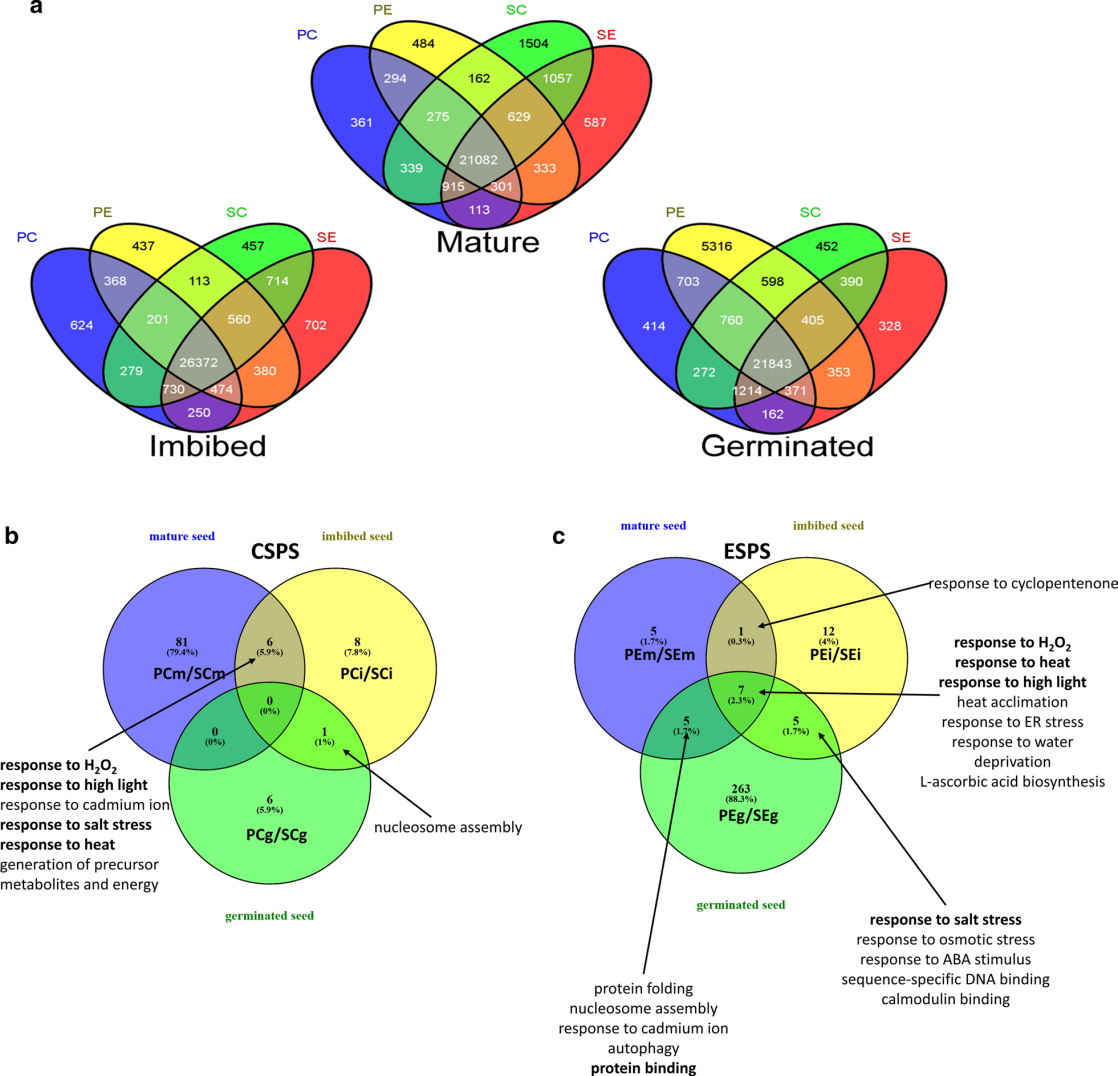
Venn diagrams of genes expression and GO term enrichment of soybean seeds during germination in response to genotype and environment. **a** Venn diagram showing total numbers of genes expressed in mature, 6-h imbibed or germinated soybean seeds. Two-letter combination indicates genotype (P = PI 587982A, S = S99-11986) and environment (C = CSPS, E = ESPS). **b** Venn diagram indicating overlap of gene ontology (GO) terms in upregulated differential genes; upregulation indicates higher level of expression in the first code listed

A core set of genes expressed was identified: (A) 21,082 in all mature seed tissues; (B) 26,372 genes expressed in all 6 h imbibed seed tissues; and (C) 21,843 genes in all germinated seed tissues (Fig. 2a, Additional file 3).

#### Differential expressed gene analysis

We utilized a Tuxedo RNAseq analysis pipeline to make 20 distinct comparisons, which can be divided into four general categories: (1) environmental effects; (2) genotypic effects; (3) the transition between mature seeds to 6-h imbibed seeds; and (4) the transition between imbibed seeds to germinated seeds (Table 1, Additional file 4).

An average of 7385 differentially expressed genes (DEGs) were detected between environments (threshold for all comparisons was q-value < 0.05). An average of 7789 DEGs were detected between genotypes. An average of 11,833 DEGs were detected between mature and 6-h imbibed seeds, across genotypes and environments (Fig. 1d). Lastly, an average of 13,344 DEGs were detected between imbibed and germinated seeds (Fig. 1d, full details are presented in Additional file 4).

#### Gene ontology enrichment

We utilized a gene ontology (GO) term enrichment tool (https://soybase.org/goslimgraphic_v2/dashboard.php) to examine lists of differentially expressed genes for the 20 comparisons (Figs. 1d, 2b, c, Additional file 5). A larger number of DEGs were found in comparisons of developmental transitions (from mature-to-imbibed or imbibed-to-germinated seeds) than within the same developmental stage (either between environments or between genotypes). Seed development/maturation under the high temperature conditions of the ESPS (Figs. 1d, 2b, Additional file 5) was associated with significant enrichment (at a threshold p-value < 0.05) for gene annotations for heat stress, response to oxidative stress and protein folding; “response to hydrogen peroxide”, “response to high light intensity”, and “response to heat” were both overrepresented and GO-term enriched in all 6 comparisons for environmental effects, with “response to wounding” overrepresented and GO term enriched in 4/6 comparisons (excepting comparisons #2, #4; comparison numbers specified in Additional file 5). The enrichment of numerous GO terms associated with abiotic stress response gives a clear indication that the mRNA pools of both genotypes are responsive to the higher temperatures of the ESPS as compared to the less stressful CSPS.

Despite this environmental response, seed mRNA pools of the heat-tolerant line were further enriched (Fig. 2b, Additional file 5) for genes with GO-terms associated with abiotic stress response [e.g. “response to high light intensity”, “response to hydrogen peroxide” and “response to heat” in 5/6 comparisons (excepting #9)” and “response to water deprivation” in 4/6 (excepting #7, #9); “response to cadmium ion” in 4/6 comparisons (excepting #9, #11); “response to salt stress” in 4/6 comparisons (excepting #9, #10)]. In addition we observed enrichment in the tolerant PI mRNA pools for protein refolding-associated GO terms: “nucleosome assembly” in 4/6 comparisons (excepting #7, #11) and “response to endoplasmic reticulum stress. The GO term “l-ascorbic acid biosynthesis” was also observed to be enriched in seed of the stress tolerant PI under the ESPS; these results are concordant our previous metabolomics study [21], which conclusively demonstrated that higher levels of ascorbate precursors were found in seeds of a heat-tolerant soybean line. Collectively, these results suggest fundamental differences exist between seed mRNA pools between the two genotypes; the more stress tolerant PI genotype is effectively “genetically primed” to more effectively manage abiotic stress as well as for higher levels of seed antioxidant compounds. This mRNA priming trend persists through seed germination and ultimately biologically translates to more efficient and effective seed germination (and in field conditions seedling emergence).

#### Weighted gene co-expression network analysis

Weighted gene co-expression network analysis (WGCNA) is a systems biology method for describing the correlation patterns among genes across samples [16]. We utilized the WGCNA package in R on FPKM data of all samples to find modules (clusters) of highly correlated expressed differential genes (≥ twofold) and a total 16 clusters were detected (Additional files 6 and 7; clusters are color coded).

Co-expressed gene clusters were then examined for overabundance of GO Biological Process terms (Additional file 8, GO Molecular Function are also provided in Additional file 9). For brevity, only Biological Process results will be discussed here. For 6/16 GO:BP clusters no significantly enriched GO terms were found (salmon, pink, purple, midnightblue, magenta, black). Several gene clusters were enriched for gene expression/chromatin remodeling (Yellow, GreenYellow, Brown) for translation/ribosome components (Yellow, Blue), mRNA splicing (Cyan, Brown) Actin/Cytoskeleton (Red, Grey) for Cell wall/Carbohydrate metabolism (Turquoise, Red). Of particular interest is the Green gene expression module, which displayed enrichment for numerous GO:BP terms annotated for abiotic stress responses (e.g. response to temperature stimulus, response to reactive oxygen species, response to salt stress, response to heat, response to stress, etc.).

#### Validation of RNAseq data using qRT-PCR

Four differentially expressed genes (Additional file 10) were selected for qRT-PCR validation of the RNAseq data. Two genes were highly expressed (*KTI*-*1*, average 86 FPKM; *HSP20*, average 856 FPKM) and two were lower expressed genes (SAM-methyltransferase, average 31 FPKM; *UDP*-*glycosyl transferase*, average 4.0 FPKM). qRT-PCR were tested via the ΔΔCt method and expressed as log2 ratios (Additional file 11). Correlations between qRT-PCR and FPKM results were robust for mature (r^2^ = 0.9729) and imbibed (r^2^ = 0.9919), but less robust for germinated samples (r^2^ = 0.6844). The high concordance between RNAseq and qRT-PCR highlights the high quality of our RNAseq dataset.

### Conclusions

In this study we provide substantial new mRNA sequencing data that defines the very early stages of soybean seed germination (mature seed > imbibed seed > germinated seed). We also contrasted two genotypes which differ in terms of tolerance to high temperature stress during seed development, which were produced under two distinct temperature stress field locations. We demonstrate that the more temperature-tolerant PI genotype is primed at the mRNA level to handle higher levels of temperature stress. In addition, we demonstrate that the PI line has faster, more efficient and more effective seed germination regardless of seed production location/environmental stress. These results highlight some of the genetic gains possible by leveraging exotic soybean germplasm as sources of novel traits in soybean breeding programs.

## Limitations

The experiment mandated a need to visually rate seeds (exposure to light) during germination on prewetted filter paper. Therefore, the transcriptomes may not completely reflect how germination of seeds in soil would proceed.

We observed poor clustering of RNAseq data for germinated seeds of the PI produced in the ESPS with other samples (PEG, Additional file 2), which is most evident in the large number of significant DEGs detected (Additional file 4).

## Additional files

**Additional file 1.** Additional field, sample, RNA sequencing and mapping methods.

**Additional file 2.** Hierarchical clustering and heatmap of differentially expressed genes.

**Additional file 3.** Cufflinks fragment per kilobase million (FPKM) results for all samples.

**Additional file 4.** Cuffdiff differentially expressed gene results.

**Additional file 5.** Gene ontology (GO) enrichment results for 20 DEG comparisons.

**Additional file 6.** Heatmap of genes analyzed by weighted gene co-expression network analysis (WGCNA, ≥ twofold difference on a log2 scale).

**Additional file 7.** Weighted gene co-expression network analysis (WGCNA) for gene function for differentially expressed genes (≥ twofold difference on a log2 scale).

**Additional file 8.** Gene ontology biological process term enrichment analysis of modules identified by weighted gene co-expression network analysis (WGCNA).

**Additional file 9.** Gene ontology molecular function term enrichment analysis of modules identified by weighted gene co-expression network analysis (WGCNA).

**Additional file 10.** Primer sequences used for qRT-PCR.

**Additional file 11.** Figure displaying correlation of qRT-PCR and RNAseq data.

## Data Availability

All sequence data obtained have been deposited in the NCBI Sequence Read Archive under project SRP090036. Analyzed datasets have also been uploaded to the SoyKB community resource (http://www.soykb.org) [22–24] and is freely available to all researchers for visualization and interactive data analysis purposes, within the “Differential Expression Suite of Tools” and gene card pages in SoyKB. PI 587982A and S99-11986 are available from the USDA-GRIN germplasm repository (https://npgsweb.ars-grin.gov/). Seed from public germplasm release DS25-1, which has heat tolerance from PI 587982A in an agronomically and yield improved line, is available by contacting Dr. Rusty Smith (Rusty.Smith@ARS.USDA.GOV). DS25-1 is also available from the USDA-GRIN germplasm repository where and can be located under the identifier PI 684675.

## Abbreviations

CSPS: conventional soybean production system
DEG: differentially expressed genes
ESPS: early soybean production system
FPKM: fragment per kilobase million
GO: gene ontology
MG: maturity group
mRNA: messenger ribonucleic acid
PI: plant introduction
qRT-PCR: quantitative real time polymerase chain reaction
RNAseq: ribonucleic acid sequencing
SG: susceptible genotype S99-11986
TG: tolerant genotype PI587982A
USDA-GRIN: United States Department of Agriculture-Germplasm Resources Information Network
WCGNA: weighted co-expressed gene network analysis

## References

[1] Heatherly LG, Spurlock SR (1999). Yield and economics of traditional and early soybean production system (ESPS) seedings in the midsouthern United States. Field Crops Res.

[2] Heatherly LG, Heatherly LG, Hodges HF (1999). Early soybean production system (ESPS). Soybean production system in the midsouth.

[3] Heatherly LG (1996). Yield and germinability of seed from irrigated and nonirrigated early- and late-planted MG IV and V soybean. Crop Sci.

[4] Hesketh JD, Myhre DL, Willey CR (1973). Temperature control of time intervals between vegetative and reproductive events in soybeans. Crop Sci.

[5] Hatfield JL, Boote KJ, Kimball BA, Ziska LH, Izaurralde RC, Ort D, Thomson AM, Wolfe D (2011). Climate impacts on agriculture: implications for crop production. Agron J.

[6] Smith JR, Mengistu A, Nelson RL, Paris RL (2008). Identification of soybean accessions with high germinability in high-temperature environments. Crop Sci.

[7] Mengistu A, Heatherly LG (2006). Planting date, irrigation, maturity group, year, and environment effects on *Phomopsis longicolla*, seed germination, and seed health rating of soybean in the early soybean production system of the midsouthern USA. Crop Prot.

[8] Hyten DL, Song Q, Zhu Y, Choi IY, Nelson RL, Costa JM, Specht JE, Shoemaker RC, Cregan PB (2006). Impacts of genetic bottlenecks on soybean genome diversity. Proc Natl Acad Sci USA.

[9] Gizlice Z, Carter TE, Burton JW (1994). Genetic base for north american public soybean cultivars released between 1947 and 1988. Crop Sci.

[10] Smith JR (2017). Soybean germplasm line DS25-1 with heat tolerance and competitive yield under heat stress.

[11] Libault M, Farmer A, Joshi T, Takahashi K, Langley RJ, Franklin LD, He J, Xu D, May G, Stacey G (2010). An integrated transcriptome atlas of the crop model *Glycine max*, and its use in comparative analyses in plants. Plant J.

[12] Severin AJ, Woody JL, Bolon Y-T, Joseph B, Diers BW, Farmer AD, Muehlbauer GJ, Nelson RT, Grant D, Specht JE, Graham MA, Cannon SB, May GD, Vance CP, Shoemaker RC (2010). RNA-Seq atlas of glycine max: a guide to the soybean transcriptome. BMC Plant Biol.

[13] Woody JL, Severin AJ, Bolon Y-T, Joseph B, Diers BW, Farmer AD, Weeks N, Muehlbauer GJ, Nelson RT, Grant D, Specht JE, Graham MA, Cannon SB, May GD, Vance CP, Shoemaker RC (2010). Gene expression patterns are correlated with genomic and genic structure in soybean. Genome.

[14] Komatsu S, Yamamoto R, Nanjo Y, Mikami Y, Yunokawa H, Sakata K (2009). A comprehensive analysis of the soybean genes and proteins expressed under flooding stress using transcriptome and proteome techniques. J Proteome Res.

[15] Le DT, Nishiyama R, Watanabe Y, Tanaka M, Seki M, Ham LH, Yamaguchi-Shinozaki K, Shinozaki K (2012). Tran L-SP: differential gene expression in soybean leaf tissues at late developmental stages under drought stress revealed by genome-wide transcriptome analysis. PLoS ONE.

[16] Langfelder P, Horvath S (2008). WGCNA: an R package for weighted correlation network analysis. BMC Bioinform.

[17] Gillman JD, Kim W-S, Krishnan HB (2015). Identification of a new soybean Kunitz trypsin inhibitor mutation and its effect on Bowman–Birk protease inhibitor content in soybean seed. J Agric Food Chem.

[18] Livak KJ, Schmittgen TD (2001). Analysis of relative gene expression data using real-time quantitative PCR and the 2(-Delta Delta C(T)) method. Methods.

[19] Libault M, Thibivilliers S, Bilgin DD, Radwan O, Benitez M, Clough SJ, Stacey G (2008). Identification of four soybean reference genes for gene expression normalization. Plant Genome.

[20] Shannon JG, Nelson RL, Wrather JA (2005). Registration of S99-11509 and S99-11986 improved soybean germplasm with diverse pedigree registration by CSSA. Crop Sci.

[21] Chebrolu KK, Fritschi FB, Ye S, Krishnan HB, Smith JR, Gillman JD (2016). Impact of heat stress during seed development on soybean seed metabolome. Metabolomics.

[22] Joshi T, Patil K, Fitzpatrick MR, Franklin LD, Yao Q, Cook JR, Wang Z, Libault M, Brechenmacher L, Valliyodan B, Wu X, Cheng J, Stacey G, Nguyen HT, Xu D (2012). Soybean knowledge base (SoyKB): a web resource for soybean translational genomics. BMC Genom.

[23] Joshi T, Wang J, Zhang H, Chen S, Zeng S, Xu B, Xu D (2017). The evolution of soybean knowledge base (SoyKB). Methods Mol Biol.

[24] Joshi T, Fitzpatrick MR, Chen S, Liu Y, Zhang H, Endacott RZ, Gaudiello EC, Stacey G, Nguyen HT, Xu D (2014). Soybean knowledge base (SoyKB): a web resource for integration of soybean translational genomics and molecular breeding. Nucleic Acids Res.

