# Supplementary material for: A seed germination transcriptomic study contrasting two soybean genotypes that differ in terms of their tolerance to the deleterious impacts of elevated temperatures during seed fill
Source: BMC Res Notes. 2019 Aug 19;12:522. doi: 10.1186/s13104-019-4559-7 (PMC6700996; doi:10.1186/s13104-019-4559-7)
Supplement: Supplementary file 1 — Additional file 1. Additional field, sample, RNA sequencing and mapping methods. [file 13104_2019_4559_MOESM1_ESM.docx]

**Details on field production of seed used in RNA-Seq study**

A MG 3 line (Huang mao bai shui dou, PI 587982A) from Sichuan, China was identified [1] to have high germinability when seed developed and matured under high temperature conditions, both in field and greenhouse testing. Compared to soybean cultivars of similar maturity grown under typical field conditions, it has low seed yield, moderate lodging, and begins to shatter shortly after maturity. MG 4 S99-11986 was collaboratively developed and released in 2004 by the University of Missouri and the University of Illinois [2]. It was derived from four accessions (PIs 297515, 290126B, 427099, and 445830), yet has high yield potential and good agronomic performance, compared to public and commercial cultivars.

For the ESPS, a single row was machine-planted each for PI 587982A and SS99-11986 at Stoneville, MS (Latitude 33.423134, Longitude -90.910876) into a Sharkey clay soil (very-fine, smectitic, thermic chromic, Epiaquert) in May 2011. Plots were 2.74 m long and 0.66 m wide and had a seeding rate of 25 seeds m^-1^. Water was supplied to plots as needed starting in June by furrow irrigation using polypipe to alleviate any potential moisture-deficit stress that could affect germinability. Tri-furilin (Aceto, Port Washington, NY, USA) was applied (1.46 liter/hectare) as a pre-emergence herbicide and Reflex (Syngenta, St. Gabriel, LA, USA) (1.17 liters/hectare) and Select Max (Valent USA, Walnut Creek, CA, USA) (0.15 liters/ hectare) were applied post-emergence. Timely cultivations and hand-weeding supplemented chemical weed control. Mustang Max (FMC Corporation, Philadelphia, PA, USA) (0.29 liter/hectare) and Acephate 97 (Tenkoz, Alpharetta, GA, USA) (0.84 kg/hectare) were applied alternately beginning in July for stink bug (*Nezara viridula*) control. In September, plots were hand-harvested shortly after maturity, threshed in a bundle thresher, and then the seed stored at 21°C and 60% relative humidity until used in germination studies.

In Missouri, both genotypes were grown in single-row, 1.5 m long plots with a 0.9 m gap and 0.76 m spacing between rows at the South Farm Agricultural Research Center (Columbia, MO, Latitude 38.908189, Longitude −92.278693, Mexico silt loam soil) in summer 2012. Field conditions were typical of soybean production in the Midwest USA. NPK Fertilizer was applied at rates appropriate per soil analyses (10.6/50/75). Two different pre‐emergent herbicides were applied: 1) Authority First (Authority First Corp, Philadelphia, PA) at a rate of 0.47 liters/hectare; and 2) Stealth at a rate of 2.3 liters/hectare (Loveland Products, Loveland, CO, USA). Post-emergent herbicides were: 1) Basagran (Arysta LifeScience North America, LLC, Cary, NC, USA) applied at 1.75 liter/hectare; 2) Ultra Blazer (UPI, King of Prussia, PA, USA) applied at 1.75 liter/hectare; and 3) Select Max (Valent Biosciences Corp., Libertyville, IL, USA) applied at 1.75 liter/hectare. At maturity, plots were hand-harvested and threshed. Seed were stored in a humidity-controlled cold (~4°C) facility until used for germination studies.

Seed were equilibrated to ~13% moisture content for two weeks in a sealed chamber with supersaturated NaCl. Seed were sterilized by soaking in 5% sodium hypochlorite for 30 sec, 15 sec in 95% ethanol, and rinsing twice in sterilized distilled water for 30 sec. Seed were moved to clean germination boxes containing two layers of blue seed germination paper (Hoffman Manufacturing, Inc. 4”x 4”) prewetted with 20 mL of sterile double distilled H_2_0.

Imbibing seed were individually weighed to determine water uptake (imbibition) and germination determined by visual examination every two hours; a seed was called germinated when the radicle had emerged.

**RNA isolation**

For each sample RNA was isolated from 5 pooled seed at one of three seed stages: 1) dry, mature seed just prior to imbibition; 2) 6-hour imbibed seed; or 3) germinated seed at the timepoint where peak germination occurred. All samples were flash frozen and stored at -80°C prior to grinding with liquid nitrogen. Total RNA was isolated from ~50 mg seed with Trizol (Invitrogen, Carlsbad, CA, USA), using on-column DNase treatment via the Direct-Zol RNA miniprep kit (Zymo, Irvine, CA, USA). One microgram DNase-treated mRNA was used to prepare libraries with a Truseq V2 RNA kit, per manufacturer’s protocols (Illumina). Samples were pooled as recommended and sequenced on three lanes of HiSeq2000 at Expression Analysis, Inc. (Durham, NC, USA).

**RNA read mapping, differential gene calling and statistical analysis**

The Illumina read cleaning, mapping, and differential expression analyses were facilitated via the RNA-Seq-Toolkit (<https://github.com/sgivan/RNA-Seq-Toolkit>) run on a CentOS7 linux operating system. A custom script was used to remove truseq adapters (AGATCGGAAGAGCACACGTCTGAACTCCAGTCAC).

Scripts from the Fastx Toolkit (<http://hannonlab.cshl.edu/fastx_toolkit/>) trimmed the 3' low quality ends (phred score < 13) bases (fastq_quality_trimmer -t 13 -l 32 -v -Q 33) and reads with <32 bases. The remaining reads were filtered to exclude those without a minimum of 90% of their bases phred score ≥13 (fastq_quality_filter -p 90 -q 13 -Q 33). Contaminants were removed using bowtie v1.0.0 [3] using PhiX - NC_001422.1 first 210 nucleotides repeated to account for circularity; polyA; polyC; gi|6164846|gb|AF184978.1|AF184978 Binary vector pCLD04541; gi|359330873|gb|AP012306.1|AP012306 Escherichia coli str. K-12 substr. MDS42 DNA. The pair of files for a sample were collated to matching sets, and orphaned reads were separated.

Reads were mapped in a splice-site aware fashion using TopHat v.2.0.9 [4]. TopHat used bowtie v2.1.0.0 to map the reads. Cuffdiff v 2.1.1 [5] was run using the multi-read correction and base composition bias correcting options. The reference genome was Glycine_max.V1.0.20.dna.toplevel.fa with the reference coordinate file Glycine_max.V1.0.20.gtf from ensemble, and additional annotation from Gmax_189 at Phytozome [6]. A false discovery rate of 0.05 was used to determine significantly differential gene expression.

Cuffdiff log2 gene expression values were used to generate a hierarchical cluster heatmap (Additional File 2) via the hclust function in R with Pearson correlations as inputs.

**References:**

1. Smith JR, Mengistu A, Nelson RL, Paris RL: **Identification of Soybean Accessions with High Germinability in High-Temperature Environments.** *Crop Sci* 2008, **48**(6):2279-2288.

2. Shannon JG, Nelson RL, Wrather JA: **Registration of S99-11509 and S99-11986 Improved Soybean Germplasm with Diverse Pedigree Registration by CSSA**. *Crop Science* 2005, **45**(4):1672-1673.

3. Langmead B: **Aligning short sequencing reads with Bowtie**. *Current protocols in bioinformatics* 2010, **Chapter 11**:Unit-11.17.

4. Trapnell C, Pachter L, Salzberg SL: **TopHat: discovering splice junctions with RNA-Seq**. *Bioinformatics (Oxford, England)* 2009, **25**(9):1105-1111.

5. Trapnell C, Roberts A, Goff L, Pertea G, Kim D, Kelley DR, Pimentel H, Salzberg SL, Rinn JL, Pachter L: **Differential gene and transcript expression analysis of RNA-seq experiments with TopHat and Cufflinks**. *Nature protocols* 2012, **7**(3):562-578.

6. Schmutz J, Cannon SB, Schlueter J, Ma J, Mitros T, Nelson W, Hyten DL: **Genome sequence of the palaeopolyploid soybean**. *Nat* 2010, **463**.
