# Supplementary figures and images for: A seed germination transcriptomic study contrasting two soybean genotypes that differ in terms of their tolerance to the deleterious impacts of elevated temperatures during seed fill
Source: BMC Res Notes. 2019 Aug 19;12:522. doi: 10.1186/s13104-019-4559-7 (PMC6700996; doi:10.1186/s13104-019-4559-7)

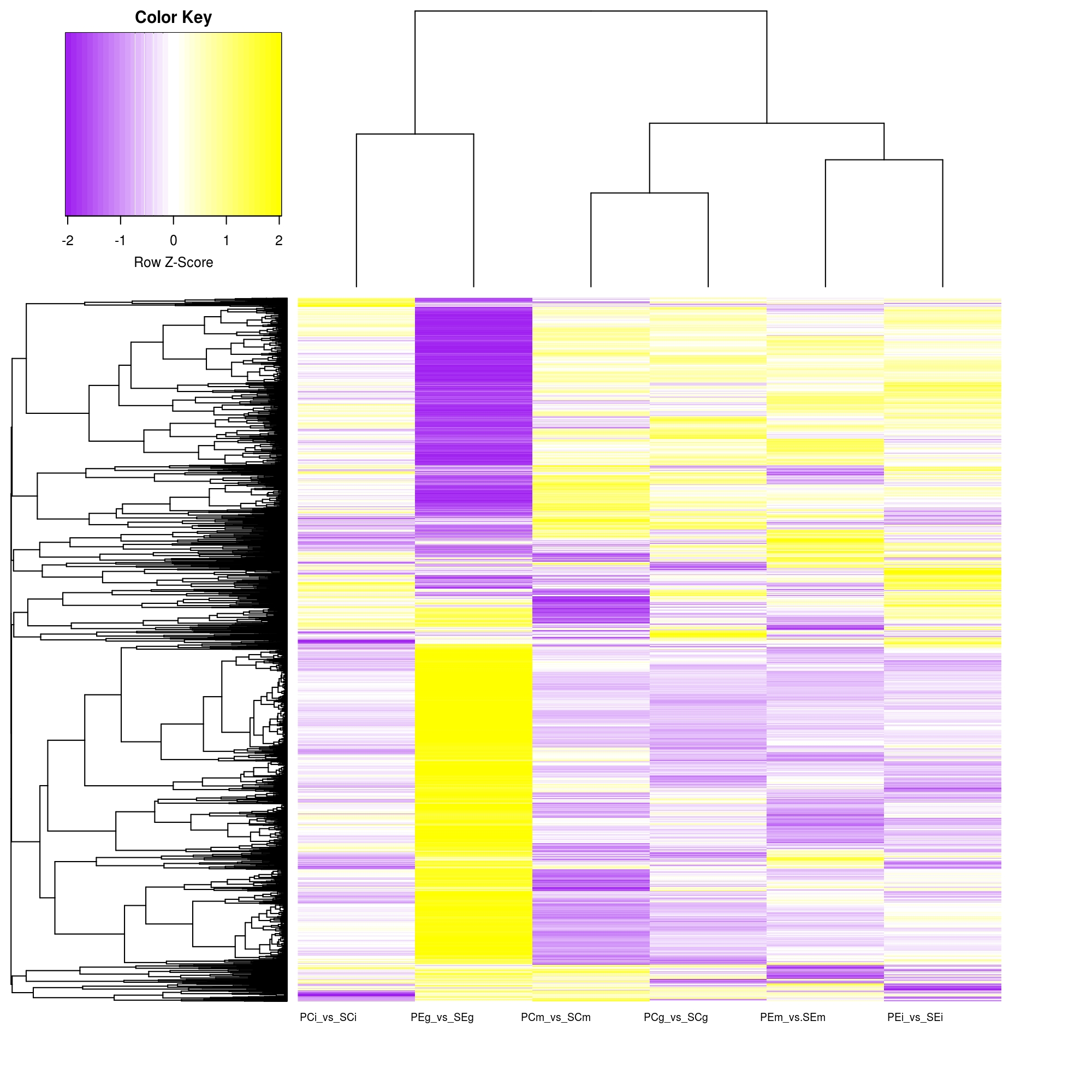

Supplement: Supplementary file 2 — Additional file 2. Hierarchical clustering and heatmap of differentially expressed genes. [file 13104_2019_4559_MOESM2_ESM.jpg]

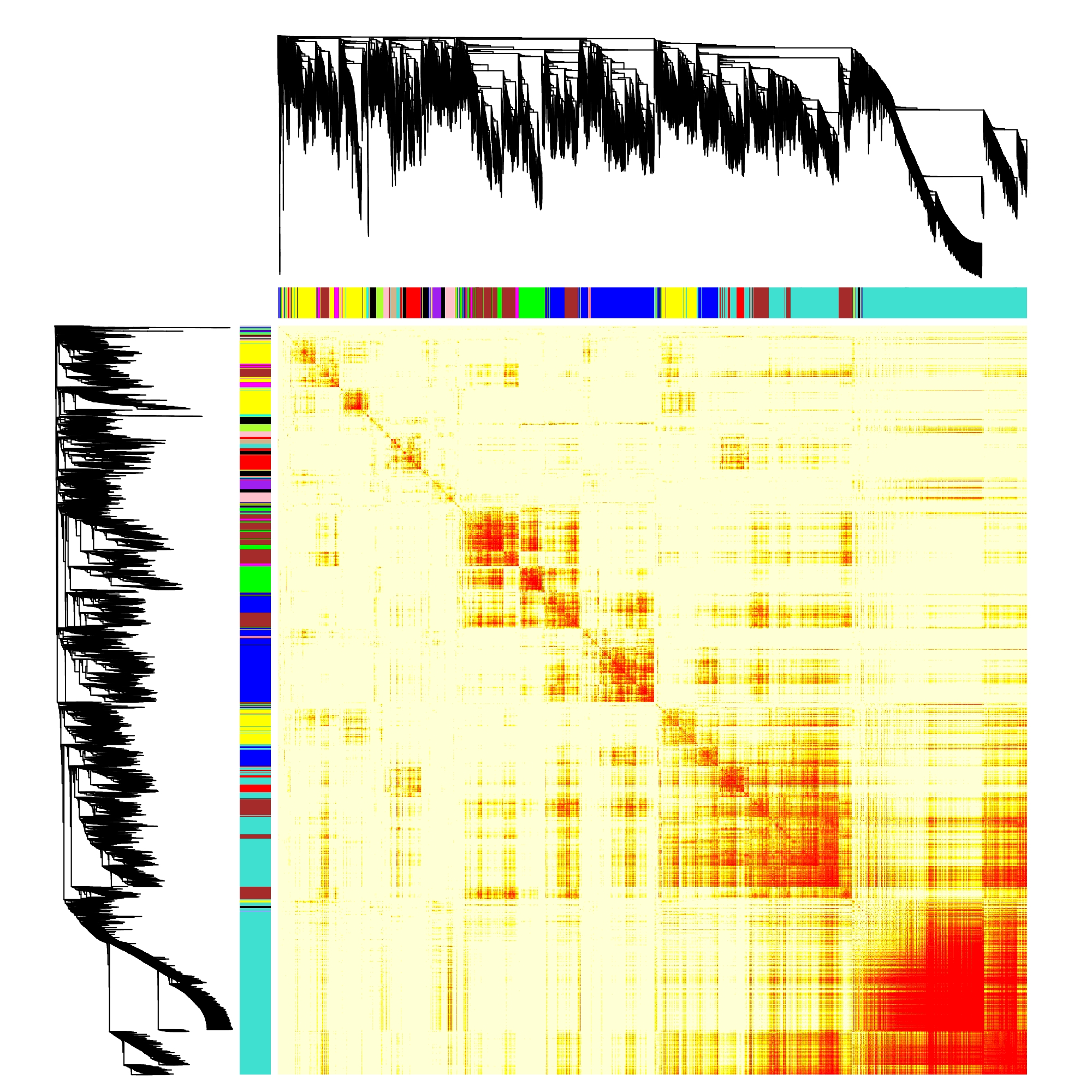

Supplement: Supplementary file 6 — Additional file 6. Heatmap of genes analyzed by weighted gene co-expression network analysis (WGCNA, ≥ twofold difference on a log2 scale). [file 13104_2019_4559_MOESM6_ESM.jpg]

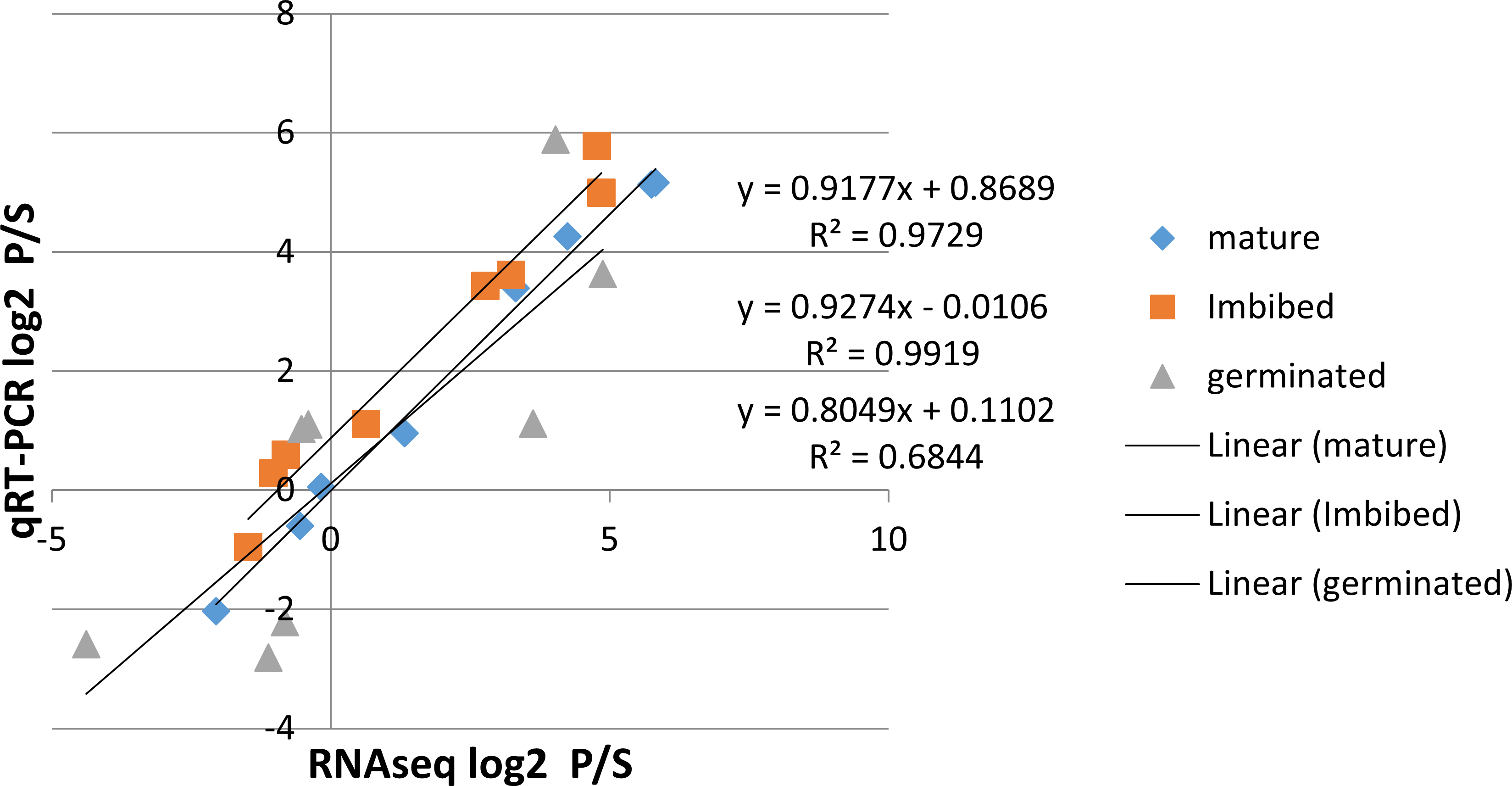

Supplement: Supplementary file 11 — Additional file 11. Figure displaying correlation of qRT-PCR and RNAseq data. [file 13104_2019_4559_MOESM11_ESM.tif]
